# The Pulse
*Page 312. Clinical Manual Series. Published by Cassell and Co.


**Published:** 1890-04-12

**Authors:** W. H. Broadbent


### THE PRACTITIONER'S BOOKSHELF.

(Books for Review should be sent to The Editor, The Lodge, Porchester Square, W.)

### THE PULSE.*—By Dr. W. H. Broadbent.

I.—(First Notice.)

In olden times, before the introduction of the sphygmograph and the thermometer, much attention was paid to the pulse as an indication of disease. But in these latter days this valuable index has stood in great danger of being neglected, or, at any rate, under estimated. It is, therefore, with much pleasure that we welcome any attempt to revive its study, and impress upon the student or practitioner the valuable lessons which are to be learned from its thorough investigation. The qualities of the pulse were the only indications of the presence of fever which physicians of the middle ages and last century possessed, and their writings show that they bestowed attentive study on its rate, rhythm, force, and fulness or size. But there could have been no true comprehension, as the work before us points out, of the significance of the pulse prior to the discovery of the circulation of the blood by Harvey in 1628. Before the time of Galen (A.D. 131) the arteries were believed to be filled with vital air, spirit, or "humour," the movements of which were thought to be independent in every part of the body, and bore no relation to the heart. Galen it was who established the fact that the arteries did not contain air, but blood, and that their rhythm was synchronous with the heart. He devoted his life to its study, and the first chapter of Dr. Broadbent's book gives a most interesting account of his work, his writings, and his views, which prevailed from A.D. 164 to the time of Harvey, a period of fourteen centuries. It is to be regretted (says Dr. Broadbent) that the clear and precise terminology of the ancients describing the different varieties and qualities of the pulse should not have been preserved. "It was a great loss to medical science that vague descriptions, such as quick, or rapid, and slow, full and bounding, firm, and the like, capable of various interpretations, took the place of definite terms employed by the early writers named. It thus became impossible to transmit by writing the knowledge gained by experience." "To-day, the study of the pulse cannot be better begun, or a better description of its variations be more clearly given than by considering separately (1) the rate or frequency of the beats; (2) the character of the individual pulsations; (3) the intensity of the beats; (4) the size of the vessel and its hardness or softness—as given by Rufus, and probably by Archigenes."

The earlier chapters are devoted mainly to physiological considerations. These form the keystone to all that follows. Thus, there are three factors which produce the pulse: 1, The action of the heart; 2, the elasticity of the larger vessels; and, 3, the resistance met with in the arterioles and capillaries. It is the first which determines the regularity and frequency of the pulse. The second tends to maintain the arterial tension at a uniform standard, just as the air cylinder of a fire-engine maintains a uniform stream of water. The varying resistance in the arterioles and capillaries is the chief factor in determining the pressure maintained in the arterial system, and the character of the individual pulsation; and it also reacts materially on the action of the heart. In the work before us, these and similar facts are clearly described and explained. There is so much that is interesting and well done, that it is hard to emphasise any. It is, however, a little doubtful if the uninitiated reader will be able fully to grasp one part, viz., the section on dicrotism, or reduplication of the pulse; that echo of the pulse or secondary wave, due to the elastic recoil of the aorta. This quality so frequently occurs under conditions attended with diminished intra-arterial pressure, that more space might with advantage have been devoted to its explanation, although full justice is done to it as a symptom in the later part of the work. The second half of the book, dealing with the clinical modifications and bearings of the pulse, is undoubtedly the most valuable. It represents the prolonged study and experience of one of the most practical physicians of our time. One space forbids our doing justice to it this week, and further notice must be postponed to a future number.

* Page 312. Clinical Manual Series. Published by Cassell and Co.

